# Changing the rules of the game

**DOI:** 10.7554/eLife.01294

**Published:** 2013-08-13

**Authors:** Dan MacLean

**Affiliations:** Sainsbury Laboratory, Norwich, United Kingdom, and an honorary senior lecturer in the Department of Computer Science, University of East Anglia, Norwich, United Kingdomdan.maclean@tsl.ac.uk

**Keywords:** cutting edge, crowdsourcing, open science, outreach, ash dieback, genomic

## Abstract

Genomics researchers have built a Facebook game that allows members of the public to join the effort to understand a disease that has killed millions of ash trees across Europe.

Crowdsourcing encompasses a wide range of activities, all of which involve getting something done using labour contributed by people who enjoy doing the thing you want to be done. It draws strength from community spirit and a sense of involvement in the project, and is made possible by the fact that social networks and mobile computing devices mean that many people are always online and able to contribute very easily.

Scientists have been quick to make use of the power of crowds in both the physical and the life sciences (see Box 1). Possibly the most audacious and visually exciting application of crowdsourcing is the Eyewire project. For Eyewire, researchers have used a laser-guided microscope to capture images of the retina, in particular the neurons within the retina, at different depths. People playing Eyewire have to colour in the neurons within these images so that, eventually, it becomes possible to combine all these two-dimensional images to make a three-dimensional model of the complex network of neurons in the retina.

Box 1. **Other examples of crowdsourcing in science**

Examples of crowdsourcing in science range from foldit, an online protein-folding game, to Galaxy zoo, which involves members of the public identifying different types of galaxies in images captured by the Hubble Space Telescope.

A novel use of Facebook for scientific crowdsourcing happened in 2011 when Devin Bloom, a PhD student at the University of Toronto Scarborough, and his colleagues needed to identify the species of 5000 fish that had been caught in the Cuyuni River in Guyana as part of a research project. Pressed for time, they uploaded photographs of the fish onto Facebook and asked other scientists and fish experts to help. Within 24 hr, all 5000 fish had been identified.

Also, the rapid release of genetic data during an outbreak of *E. coli* in Germany in 2011 was instrumental in the rapid identification of the strain responsible for the outbreak because it allowed bacterial genomicists from around the world to contribute analyses of the data.

More recently, scientists from the Wellcome Trust Sanger Institute mentored budding game developers in a short coding competition to produce a game based on genomics and genetics. And Cancer Research UK is collaborating with Amazon, Facebook and Google to run a ‘GameJam’ to design and develop a mobile phone game that would analyse genetic data as part of the effort to speed up the development of improved treatments for cancer.

The usefulness of using crowdsourcing to do genetics research has been demonstrated by the results of the Phylo project. Phylo is a game in which the four bases in DNA are represented by blocks of different colour: the aim of the game is to move groups of blocks to the left and right in order to find the best possible alignment of groups with each other. Or, in the language of bioinformatics, to work on a computationally intractable problem called ‘multiple sequence alignment’. The game has provided the Phylo team with many thousands of improved solutions to this problem for genes related to disease in humans and 43 other vertebrate species (see Kawrykow et al., 2012; note that ‘Phylo players’ are listed as an author on this paper).

## More than a game

Both of these games require a low amount of investment: from the players’ point of view, they can be dipped into in a few spare minutes and played for their intrinsic reward as a game and still return useful results. However, this minor effort per player does not mean that the results are trivial: rather, there are certain types of problems that the human brain and eye can solve much faster than a computer can solve them.

For problems that require a high level of investment in time and application, and perhaps specialist knowledge from a different field, incentives beyond those provided by a game have been provided. For example, Karim Lakhani of Harvard Business School and co-workers ran a prize-based contest in which they invited algorithmic scientists from outside of biomedicine to develop algorithms to annotate (i.e. correctly label) genetic recombination in human T cells (Lakhani et al., 2013). The advances sought here were substantially technical, and the contest brought to the fore a variety of techniques that had not previously been tried by the computational biology community: moreover, some of the entries achieved algorithmic speeds that were faster than existing techniques by a factor of over 1000, and some entries achieved accuracies that were close to the theoretical maximum for the test data set.

A crucial component of this approach, apart from the promise of a prize, was the appeal to a pre-existing community. The contest was launched through the TopCoder.com website, a community of more than 500,000 coders who regularly compete to solve coding problems. To reach this community effectively, the genetics problem the researchers were interested in was recast as an algorithmic problem that was better suited to the target audience.

When we want to make use of crowds to do specialist analyses, one of the challenges is to accommodate their non-expert status (or, in the previous example, their other-expert status) by translating the problem into a form that is readily understandable by the crowd. Also, in many cases non-experts will not appreciate the value of the thing they are working towards, so other goals and rewards must be wrapped around the activity. Game interfaces are able to mix these three factors—a problem that can be understood, a goal and a reward—together. This is the approach that the OpenAshDieBack project—run by researchers at the Sainsbury Laboratory, the John Innes Centre and the Genome Analysis Centre, all in Norwich in the UK—is now taking to gain a better understanding of ash dieback, a fungal disease that has killed millions of ash trees (*Fraxinus excelsior*) across Europe.

## An open approach to genomics

Ash dieback is caused by a fungus, *Chalara fraxinea*, that emerged in Poland in the early 1990s and spread west across Europe. There is no known treatment for the disease, so when it reached the UK in 2012, the author and colleagues set up the OpenAshDieBack project to rapidly generate genomic sequence data for *C. fraxinea* and release these data directly to the scientific community (even before we had a chance to analyse them ourselves). This crowdsourcing approach enabled us to gather analyses from scientific experts around the globe, and we now have a variety of new genomic resources, including draft genome assemblies of both *F. excelsior* and *C. fraxinea*.

To encourage input from beyond the scientific community we have now developed a game interface that allows non-geneticists and non-scientists to contribute to our genomic analyses by analysing genetic variants within Facebook. The game, which is called Fraxinus, is not merely an exercise in outreach, it is designed to return data that are useful to scientists.

The process of preparing a genome sequence is complex because genomes are extremely long and because existing sequencing techniques have various shortcomings. The genome of *C. fraxinea* is 63 million bases long and the genome of the ash tree is even longer (approximately 954 million bases). The biochemical process we use to sequence these genomes can give us fragments of less than 200 bases: this means that we have millions of fragments (or ‘reads’) for each genome, and we must assemble these reads together into longer contiguous sections in order to understand the genetic code of that organism.

Individuals from the same species will have DNA sequences that are almost identical. However, the differences between them are important because they contribute to differences in, for example, the ability to cause disease (for pathogens) or the susceptibility to disease (for trees). Therefore, by comparing the DNA sequences from different samples with a reference genome for that species, we should be able to identify the genetic variations that make some strains of *C. fraxinea* so deadly and/or the variations that make some trees resistant to ash dieback.

Common variants include a simple change in the identity of a base—such as an adenine (A) being replaced by a thymine (T)—or the insertion/deletion of a base compared with the reference (a variant called an indel). A lot of research has gone into developing computer programs that can identify these variants automatically, but computational approaches have some significant shortcomings, especially when identifying indels in certain types of genomes, such as genomes that are highly repetitive or contain a lot of cytosine (C) and guanine (G) bases. In these situations, the visual acuity and excellent pattern recognition skills of humans can be invaluable and can help us to find these elusive and potentially crucial variants.

## Game on for genomics

The basic idea of Fraxinus is to compare reads from different samples (which could be pathogens or trees) with the relevant reference genome to identify important variants. To make this challenge manageable, we have selected locations within the reference genome that we know (based on a preliminary analysis of the data) are likely to contain genetic variants and included the bases on either side of these locations to make target patterns that contain a total of 21 bases. We have then extracted all the reads that overlap to some extent with each target pattern. To begin with, the game will only contain data on the pathogens, but the game could be played with data from any species, and we plan to include data on the trees within the next few weeks.

**Figure 1.**
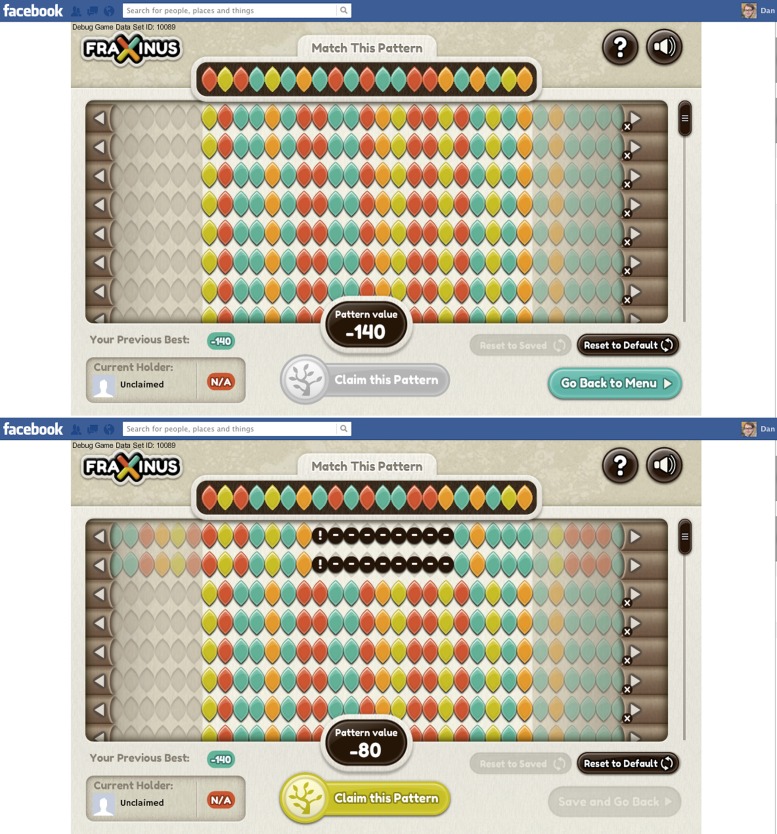
Fraxinus in Facebook. The top screenshot shows the start of the game. The target pattern is at the top of the screen and the aim of the game is to align the read patterns with this target. The target pattern is always a 21-base stretch of DNA from a reference genome that is likely to contain a genetic variant; in Fraxinus we are starting with targets taken from a reference genome for *C. fraxinea*, the pathogen that causes ash dieback disease, but the targets can be chosen from any reference genome. The read patterns used in the game can contain up to 76 bases and, in this case, are taken from samples of an interesting strain of *C. fraxinea*. The arrow buttons allow each read pattern to be moved left or right, while individual leaves (bases) can be moved, deleted or inserted with click-and-drag movements. The pattern value at the bottom of the screen indicates the player’s score. The bottom screenshot shows the game after the player has shifted the top two reads 14 bases to the left to match the seven bases on the left of the target, and then introduced a 9-base insertion (black circles) to match another three bases towards the right of the target: these moves increase the pattern value to −80. (Note that zero is not the maximum possible score.)

In the game the A, C, G and T bases within both the target and read patterns are represented by coloured leaf shapes (see Figure 1). The aim of the game, for the player, is to align the read patterns with the target pattern: the better the alignment between these two patterns, the higher the player’s score. The player can improve the alignment—and, therefore, his or her score—by shifting the whole read pattern, and then hiding individual leaves (to represent a deletion) or introducing a space between two leaves (an insertion). And if there already is a space between two leaves, it can be increased in size or removed.

The player’s score is recalculated after every action, and play ends when the player decides that he or she cannot improve the score any further. If the player’s score is the highest for that target pattern, the player ‘owns’ the pattern. The player can then either tackle a target pattern that is not owned by another player, or attempt to ‘steal’ a target pattern owned by someone else by getting a higher score for it. Players know that the alignments they create will be stored by the scientists running the game and that their names will also be stored (if they consent) so that they can be credited for their contribution to science.

Parts of the genome of *C. fraxinea* are rich in G and C bases, which makes it difficult to identify variants with computational techniques, so there is a good chance that the game will supply us with a rich supply of new variants to analyse. With more accurate lists of genetic variants we will be much better placed to assess genetic variability between strains and to carry out experiments to associate particular organismal traits (such as the ability to cause or withstand infection) with their genetic root. Ultimately this will help us to breed trees that are resistant to ash dieback (or other diseases), to identify treatments, and to plan for better management of our woodlands and tree stocks.

By embedding Fraxinus in the Facebook platform, we hope to encourage competition between players and other members of their social network, as this will lead to multiple independent analyses of the same data and to more accurate results. However, anyone attempting to do something similar should be aware that game design is difficult, and it requires input from experienced software engineers, gameplay experts and graphic designers. (We worked with a company called Team Cooper to help us develop Fraxinus.) A key challenge is to bridge the gap between the players and the scientists—if the game is not exciting to play, the project will fail no matter how exciting the science is to the scientists.

We think that with Fraxinus we have created a game that exists in a happy middle ground as a game platform that effectively and simply encapsulates the problem we need to solve, that is enjoyable and competitive for the player and, we hope, can provide us with the results we need.
